# Modeling-Based Investigation of the Effect of Noise in Cellular Systems

**DOI:** 10.3389/fmolb.2018.00034

**Published:** 2018-04-12

**Authors:** Didier Gonze, Claude Gérard, Benjamin Wacquier, Aurore Woller, Alen Tosenberger, Albert Goldbeter, Geneviève Dupont

**Affiliations:** ^1^Unité de Chronobiologie Théorique, Faculté des Sciences, Université Libre de Bruxelles, Brussels, Belgium; ^2^de Duve Institute (LPAD Group), Université Catholique de Louvain, Brussels, Belgium

**Keywords:** stochastic simulation, molecular noise, robustness, circadian clock, cell cycle, calcium signaling, embryonic development

## Abstract

Noise is pervasive in cellular biology and inevitably affects the dynamics of cellular processes. Biological systems have developed regulatory mechanisms to ensure robustness with respect to noise or to take advantage of stochasticity. We review here, through a couple of selected examples, some insights on possible robustness factors and constructive roles of noise provided by computational modeling. In particular, we focus on (1) factors that likely contribute to the robustness of oscillatory processes such as the circadian clocks and the cell cycle, (2) how reliable coding/decoding of calcium-mediated signaling could be achieved in presence of noise and, in some cases, enhanced through stochastic resonance, and (3) how embryonic cell differentiation processes can exploit stochasticity to create heterogeneity in a population of identical cells.

## Introduction

Noise arises at all levels of biological organization and inevitably affects the dynamics of biological systems. Cellular processes, ranging from gene expression to developmental pathways, signaling, cell division, and circadian rhythms are all exposed to noise, but the way they cope with it varies from one case to another, and it is therefore not straightforward to predict the effect of noise. Noise tends to compromise the precision and the timing of molecular events, and is thus often seen as a nuisance (Kaern et al., 2005). However, in some contexts, it may actively contribute to biological functions (Eldar and Elowitz, 2010). Complementary to the experiments, modeling-based approaches constitute a powerful tool to explore the impact of noise and its possible role in cellular dynamics.

Noise originates from multiple sources. Molecular noise, sometimes referred to as intrinsic noise (Elowitz et al., 2002), arises from the low number of molecules and the intrinsic stochastic nature of biochemical reactions in the cell. Extrinsic sources of noise include small variations in microenvironmental conditions (temperature, pH, concentration of diffusive ligands in the inter-cellular medium), as well as inevitable cell-to-cell variability in factors like the number of RNA polymerases, ribosomes, proteases, etc. All these sources of variability result in small differences in kinetic parameters (transcription/translation rates, etc.). Moreover, cells divide and move. At division, molecules are not equally distributed into the two daughter cells and the surrounding environment is continuously changing. In particular, cells do not remain in contact with the same cells and this remixing of the neighborhood also contributes to stochasticity.

When noise impairs the proper dynamics of a cellular process, it is expected that regulatory mechanisms have evolved to counteract this negative effect of noise. For example, to ensure a proper adaptation to the regular light-dark cycle, the circadian clock must be quite robust despite the limited number of mRNA and protein molecules involved in the clockwork (Barkai and Leibler, 2000). Similarly, a tight control of cell proliferation can only be achieved by a reliable regulation of the cell cycle. One can thus expect that the complex molecular mechanisms underlying these processes enable robust oscillations with respect to noise.

Cell signaling is also subject to noise. It involves coding and decoding messages, and a reliable communication between cells and between cells and the environment is needed for a proper function (Kholodenko, 2006). A prototypical example of cell signaling, involving intracellular Ca^2+^ dynamics (Berridge, 2009), is clearly random at the sub-cellular level but displays quite regular Ca^2+^ spiking at the level of the whole cell. This example allows for the observation of the passage from a noise-driven irregular behavior to a more or less regular spiking, that could correspond to deterministic oscillations perturbed by noise upon increasing the level of stimulation. Interestingly, even when stochasticity plays a predominant role, reliable frequency encoding is possible (Thurley et al., 2014). Besides the level of stimulation, Ca^2+^ transfer between the various intracellular organelles also affects Ca^2+^ dynamics and the noise on the period of Ca^2+^ oscillations. Given that entry of Ca^2+^ into mitochondria stimulates ATP synthesis, this noise has physiological consequences on metabolism (Szabadkai and Duchen, 2008).

Additionally, noise is responsible for cell-to-cell variability and this cellular heterogeneity may have important implications for the development of multicellular organisms and for the fitness of microbial colonies (Balázsi et al., 2011; Sanchez et al., 2013). In multicellular organisms, the process of cell differentiation, whereby identical cells divide, and adopt different fates, may exploit noise to create cellular heterogeneity. Such heterogeneity is required to initiate different developmental pathways. Yet, regulatory mechanisms must ensure a proper partition of the cells in each state and possibly their spatial organization. In micro-organisms, the role of stochastic gene expression in phenotypic variability and its ability to lead the cells in one or another developmental pathway was described theoretically early on (see for example Arkin et al., 1998), but the adaptative role of such phenotypic heterogeneity, often postulated, was experimentally demonstrated much later (Acar et al., 2008).

Theoretical studies have for long emphasized the importance of stochastic fluctuations in biological systems (Delbrück, 1940; Monod, 1971; Spudich and Koshland, 1976). Stochastic models for gene expression and enzymatic reactions were already published in the sixties (Jachimowski et al., 1964; Asai and Morales, 1965; Singh, 1969; Blum, 1974). More recently, questions have been raised as to the impact of noise on complex signaling and regulatory networks underlying highly organized behaviors such as oscillations and multi-stability. In the absence of noise, oscillatory processes are often modeled by limit cycle oscillators. These models allow to identify requirements to generate self-sustained oscillations and shed light on the roles of regulatory circuits (Goldbeter et al., 2012). Cell fate determination and differentiation are viewed as reflecting the possibility for the cell to evolve to one or another stable steady state, which may co-exist, and are thus explained in terms of multistability (Huang et al., 2005). Modeling studies highlight the conditions for the emergence of bistability, often associated to hysteresis, and the molecular mechanisms allowing a cell to evolve to one or another state. Deterministic models, relying on non-linear ordinary differential equations (ODE) and bifurcation theory, are appropriate to characterize these design principles and make testable predictions, but they do not account for noise and its effect on the dynamics. However, advances in biotechnologies now allow measuring noise in single cells. To take these data into account and investigate the effect of noise in a series of ODE-based models for oscillations or multi-stability, modelers can take profit of the high computational power to perform CPU-consuming stochastic simulations based, for example, on the algorithm proposed by Gillespie (1977). This method is described and illustrated in the Supplementary Information, where we also provide the references to the original publications for the results shown here.

We illustrate here, through a couple of selected examples, how modeling and numerical simulations can be used to assess the role of noise in the dynamics of biological systems. In particular, we focus on factors that likely contribute to the robustness of circadian clocks and the cell cycle with respect to noise, on how reliable calcium-mediated signaling coding/decoding are achieved—and even enhanced—in presence of noise, and on how embryonic cell differentiation processes can exploit stochasticity.

## Robust circadian rhythms from sloppy individual cellular oscillators

Circadian rhythms, characterized by an endogenous period around 24 h, are responsible for the daily timing of physiological functions. The core molecular mechanism of these rhythms relies on a negative transcriptional feedback loop. Theoretical models for circadian rhythms based on such control mechanisms have been proposed soon after the identification of the first clock genes (Goldbeter, 1995; Ruoff et al., 1996; Leloup et al., 1999). These models have been used to study temperature compensation (Ruoff et al., 1996) or entrainment by light-dark cycles (Leloup et al., 1999). Extensions of these models incorporating multiple regulations have been used to investigate the role of the regulatory feedback loops in the generation of self-sustained circadian oscillations and to explore the links between the circadian clock and physiological disorders (Leloup and Goldbeter, 2003; Becker-Weimann et al., 2004).

Barkai and Leibler (2000) raised the question of the robustness of such circuit in presence of molecular noise—and thereby of the validity of the deterministic models. This prompted the development of stochastic models for circadian clocks. Numerical simulations of these models (using algorithms such as the one proposed by Gillespie, 1977) led to the identification of several factors that enhance the robustness of the oscillations. These factors include the degree of cooperativity of gene repression (non-linearity) and the rate of binding-unbinding of transcription factors to the gene promoter (Gonze et al., 2002; Forger and Peskin, 2005). Periodic entrainment by the light-dark cycles was also shown to stabilize the phase of the oscillations with respect to molecular noise (Gonze et al., 2002).

In mammals, the central pacemaker is located in the suprachiasmatic nucleus (SCN) of the hypothalamus. The SCN receives light information from the retina and generates a robust, entrainable rhythm, transmitted to peripheral organs. Oscillations in the SCN occur at the level of single neurons while inter-cellular coupling, relying on the periodic release of neurotransmitters, ensures the synchronization between individual cells (Yamaguchi et al., 2003). Single-cell recording of clock gene luciferase reporter showed that dispersed cells can maintain circadian oscillations over several 24-h cycles but display a large variability of period and amplitude (Honma et al., 1998; Yamaguchi et al., 2003; Webb et al., 2009).

The idea that the circadian clockwork may be composed of a collection of sloppy oscillators made robust upon inter-cellular coupling dates back to Enright (1980) and appears to be supported by experimental observations in the SCN (Herzog et al., 2004). Indeed cycle-to-cycle period variability appears 10 times greater in dispersed SCN cells than in connected cells in the intact SCN tissue. Similarly, treatments with agents that impair neuronal coupling, result in sloppy circadian rhythms of clock gene expression in many cells (Yamaguchi et al., 2003).

**Figure 1:**
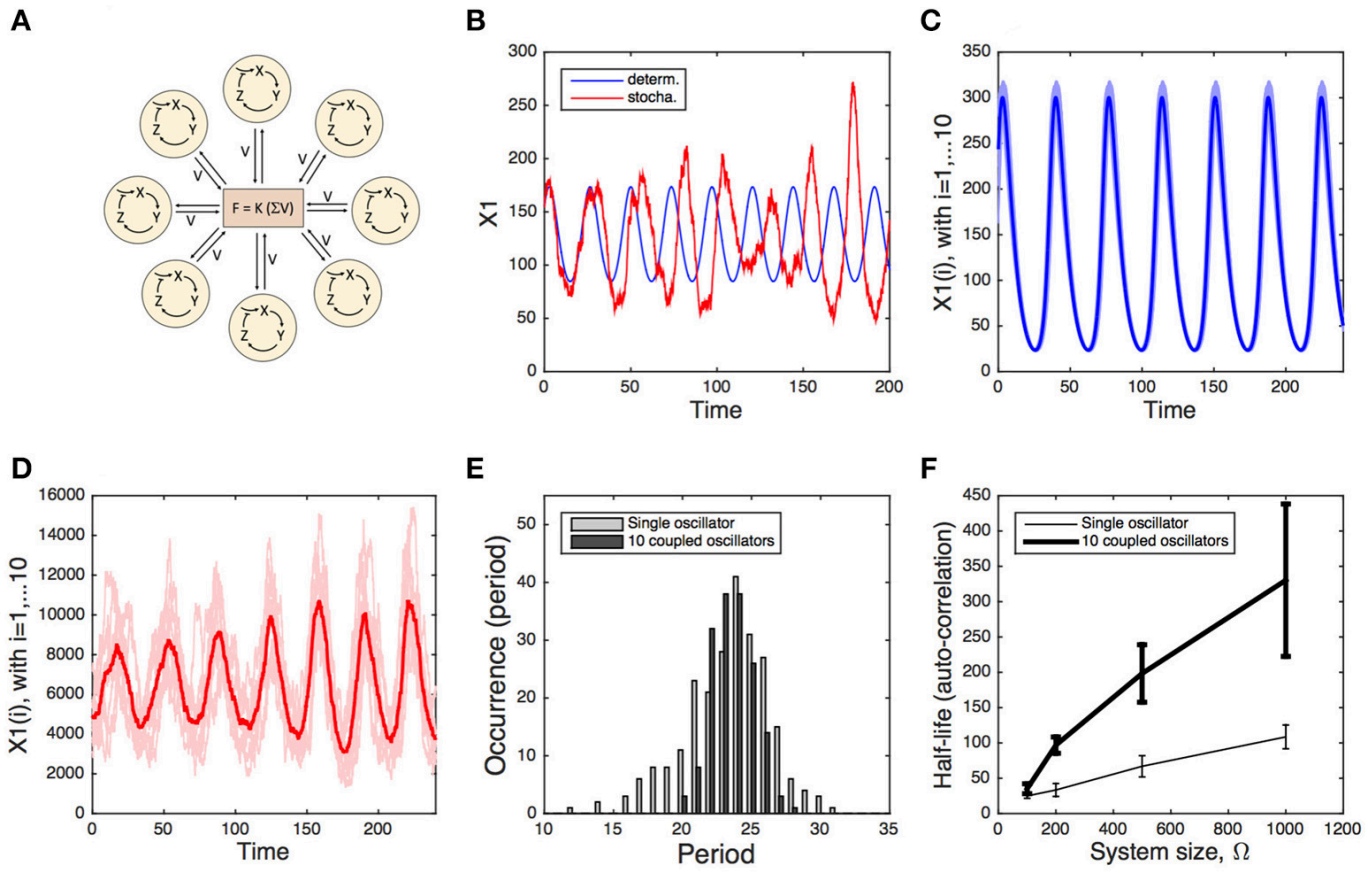
Deterministic vs. stochastic simulation of a system of coupled circadian oscillators. **(A)** Scheme of the model. Each oscillator is modeled by a 3-variable Goodwin-like model. The oscillators are coupled through the mean field obtained by the average concentration of the clock-controlled release of a neurotransmitter (see Gonze et al., 2005 for the description and deterministic analysis of the model). **(B)** Deterministic (blue) and stochastic (red) oscillations of the single-oscillator model. **(C)** Deterministic oscillations of 10 coupled oscillators, displaying 10% of variability in their free-running period. **(D)** Stochastic oscillations of the same 10 coupled oscillators. Stochastic simulations in **(B,D)** have been carried out for a system size Ω = 500. **(E)** Period distribution of the single oscillator (gray) and the 10 coupled oscillators (Ω = 500, black). **(F)** Half-life of the auto-correlation as a function of the system size Ω, determined for the single oscillator (thin curve) and the 10 coupled oscillators (thick curve). The error bars denote the standard deviation over 10 simulations. Stochastic simulations have been performed using the Gillespie algorithm (see Supplementary Information), as described in Gonze et al. (2002) and in Gonze and Goldbeter (2006).

Mathematical modeling showed that efficient synchronization can be achieved by the coupling of oscillators via the periodic release of neurotransmitters (Gonze et al., 2005). Stochastic simulations of this system further support the idea that inter-cellular coupling can significantly contribute to the robustness of the overall network (Figure 1). Stochastic simulations of individual oscillators show that they undergo rapid phase diffusion, resulting in a fast desynchronization with respect to the corresponding deterministic time series (Figure 1B). When 10 oscillators are coupled, they quickly synchronize and display an increase in amplitude, even in the presence of 10% variability in their free running periods (Figure 1C). Stochastic simulations of this coupled system suggest that, despite some inter-cellular variability, the average signal of the coupled system is more robust (Figure 1D) than the one of the individual cells, as attested by the narrower distribution of periods (Figure 1E) and by the increased half-life of the auto-correlation (Figure 1F). Similar conclusions have been reached with a model of coupled oscillators reflecting the syncytial coupling of circadian oscillators in *Neurospora* (Gonze and Goldbeter, 2006).

In the above model, the individual oscillators display self-sustained (limit cycle) oscillations. Similar results are obtained when individual oscillators are parameterized to yield damped oscillations (not shown). Thus, intercellular coupling can induce robust, self-sustained oscillations. Whether the noise alone is able to convert damped, sloppy circadian oscillators into self-sustained oscillations was addressed by Westermark et al. (2009). These authors analyzed experimental time series and compared the observed dynamics with the theoretical predictions for two scenarios: noisy self-sustained oscillations vs. noise-driven damped oscillations. This analysis however did not allow a clear discrimination between the two cases, both being plausible.

In a combined experimental-modeling study, Ko et al. (2010) analyzed the effect of loss-of-function *Bmal1* mutant in the dynamics of the SCN. *Bmal1* is a core clock gene and its knock-out results in a loss of circadian rhythmicity in individual cells. Remarkably, a clear rhythm—although noisy—emerged from the SCN network even in presence of this mutation. These observations were also reproduced by a mathematical model. They suggest that the clock network can exploit noise to compensate for the loss-of-function mutation.

## Positive feedbacks as a noise-reduction mechanism in the cell cycle

The cell division process plays a major role in unicellular and multicellular organisms. In the latter, it drives the development from fertilized eggs into mature organisms. In the mature organism, cell division allows the replacement of cells that die due to natural causes or external damage. Thus, the cell cycle plays a crucial role in the development of living organisms both in normal and disease conditions. The cell cycle is composed of four different phases: G1, S (DNA replication), G2, and M (mitosis) and is controlled by a network of cyclin-dependent kinases (CDK) whose activities drive the progression along the successive phases of the cell cycle (Morgan, 2007).

Computational models were initially proposed for the dynamics of the cell cycle in frog embryos (Goldbeter, 1991; Tyson, 1991; Novak and Tyson, 1993), where the core mechanism of the cell cycle relies on a negative feedback exerted by a CDK on itself (Goldbeter, 1991). Afterwards, more detailed models for the yeast cell cycle were proposed (Novak et al., 2001; Chen et al., 2004). In fission yeast, the ordered progression through the successive phases of the cell cycle is controlled by a single CDK, Cdc2, required for both the G1/S and G2/M transitions. DNA replication and mitosis are triggered by association of Cdc2 with the B-type cyclins Cig2 and Cdc13, respectively. Each cell cycle transition is controlled by positive feedback (PF) loops ensuring robust and ordered progression along the different cell cycle phases (Sha et al., 2003; Novak et al., 2007).

In higher eukaryotes and mammals, the cell cycle is driven by a complex network of CDK composed of intertwined negative and positive feedback loops. The activities of cyclin D/Cdk4-6 and cyclin E/Cdk2 ensure progression in G1 and promote G1/S transition, respectively. Cyclin A/Cdk2 drives progression in S and G2, while the activity of cyclin B/Cdk1 triggers the G2/M transition. Detailed models were proposed to account for the dynamics of the mammalian cell cycle (Novak and Tyson, 2004; Swat et al., 2004; Gérard and Goldbeter, 2009). Again, each cell cycle transition such as the G1/S (Barr et al., 2016), the G2/M (Pomerening et al., 2003) or the anaphase-metaphase checkpoint during mitosis (He et al., 2011) is controlled by PF loops ensuring a robust, ordered, progression through the cell cycle (Han et al., 2005; Verdugo et al., 2013).

Numerous models were proposed to assess the impact of molecular noise on the dynamics of the cell cycle in yeast (Tyson, 1989; Sveiczer et al., 2001; Kar et al., 2009; Ball et al., 2011; Liu et al., 2012; Barik et al., 2016), in mammals (Gérard et al., 2012), or in frog embryos (Gonze and Hafner, 2010).

The stochastic study by Kar et al. (2009) reproduces many characteristic features of the yeast cell cycle, despite the very low abundance of mRNAs molecules (~1 mRNA molecule per cell for each expressed gene). This agreement is obtained when assuming that some specific mRNAs have very short half-lives—less than 1 min.

**Figure 2:**
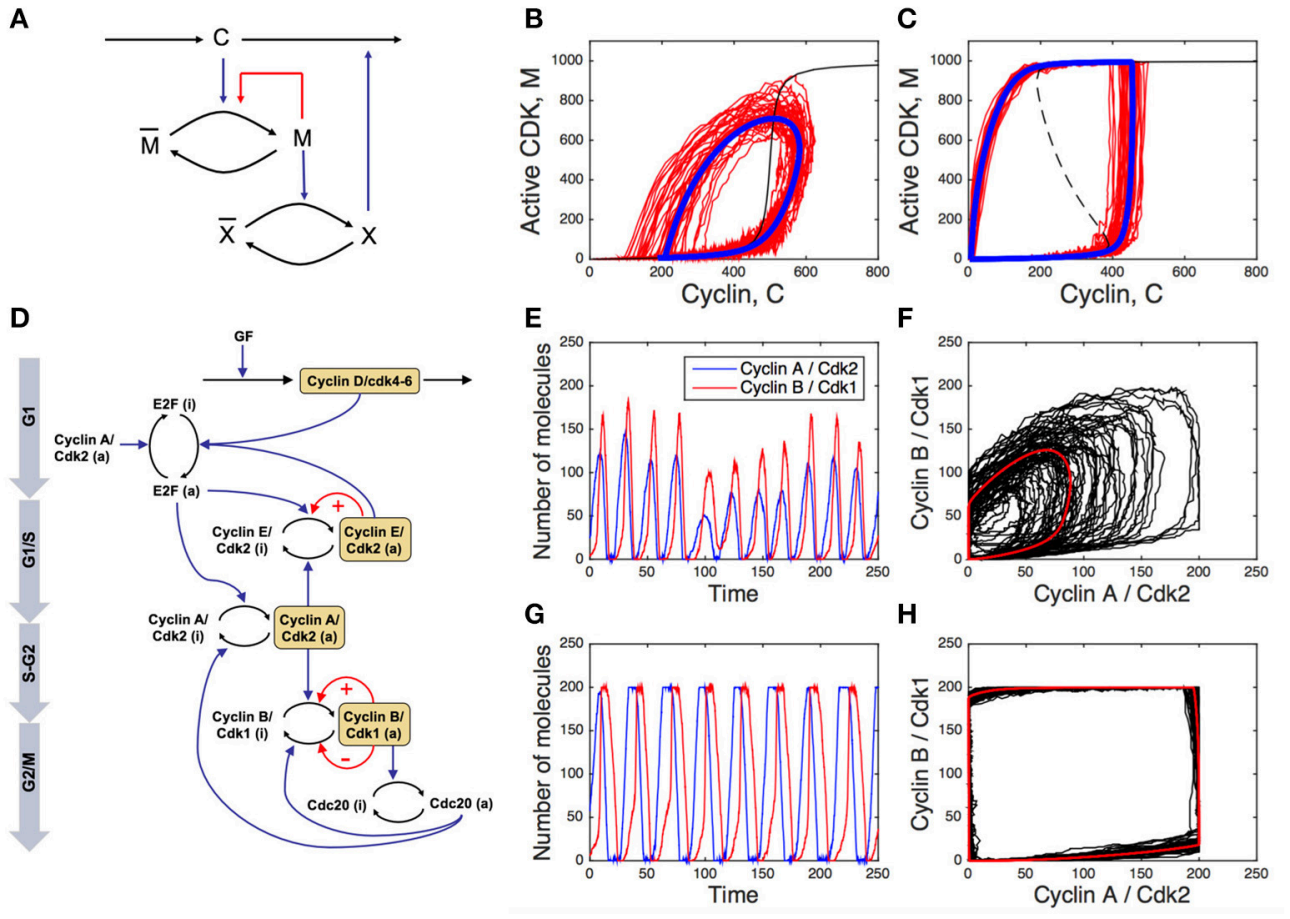
Impact of positive feedback loops on the cell cycle robustness. **(A)** Scheme of a toy model for embryonic cell cycle (C, cyclin; M, CDK; X, protease), with positive feedback (PF) loop denoted by the red arrow. **(B,C)** Deterministic (blue curves) and stochastic (red curves) limit cycle obtained without **(B)** or with **(C)** PF loop (see Gonze and Hafner (2010) for details). The dashed, black curve is the steady state of M as a function of C obtained when C is kept constant. **(D)** Scheme of the skeleton model for the mammalian cell cycle. The red arrows indicated the PFs. **(E,G)** Stochastic temporal evolution of cyclin E/CDK2 and cyclin B/CDK1 in the absence **(E)** or presence **(G)** of PF driving the G1/S and G2/M transitions of the cell cycle. **(F,H)** Deterministic (red curves) and stochastic (black curves) limit cycle oscillations in the cyclin B/CDK1 vs. cyclin A/CDK2 plot in the absence **(F)** or presence **(H)** of PF loops. See Gérard et al. (2012) for details.

In addition to the possible tuning of kinetic parameters, the regulatory network may also have evolved to increase its resistance to noise. This idea was tested in a toy model for the embryonic cell cycle (Gonze and Hafner, 2010; Figures 2A–C). This model includes the cyclin (C), the CDK (M) which is activated by the cyclin, and a protease X which is activated by the CDK and degrades the cyclin, thus closing the negative circuit. The PF is introduced through the auto-activation of the CDK. Stochastic simulations of this 3-variable system indicates that the presence of positive feedback reinforces the dynamics of the CDK network in presence of molecular fluctuations, as observed when comparing the deterministic and stochastic limit cycles in the absence (Figure 2B) or presence of a PF loop on CDK activity (Figure 2C). This model also provides an explanation for the gain in robustness. The positive feedback introduces underlying bistability and hysteresis. Indeed, the steady state of CDK as a function of the cyclin (taken constant) goes from a sigmoidal shape (in absence of positive circuit) to an S-shape (in the presence of a positive circuit). In the latter case, when the negative circuit is at work, the cyclin oscillates, switches back and forth over the bistable region and induces fast and well-marked transitions between the inactive and the fully active forms of the CDK. This fast-slow dynamics dampens the effect of molecular noise. A systematic parameter screening confirms that this gain in robustness does not depend on parameter values (Gonze and Hafner, 2010).

The role of multiple, redundant, PF loops in the robustness of the oscillatory dynamics of the CDK network was also studied in more detailed cell cycle models (Domingo-Sananes and Novak, 2010; Gérard et al., 2012). The scheme in Figure 2D represents a skeleton model for the CDK network driving the mammalian cell cycle (Gérard et al., 2012). A PF loop is present at the G1/S transition through the mutual activation between CDK2 and its phosphatase CDC25, while two redundant PF loops regulate the core of the G2/M transition of the cell cycle, through the mutual activation between CDK1 and its phosphatase CDC25, and the mutual inhibition between CDK1 and the kinase WEE1. The temporal evolution of cyclin E/CDK2 and cyclin B/CDK1 is more robust to stochastic fluctuations in the presence than in the absence of PF loops (compare Figures 2E,G). In addition, the presence of PF loops considerably enlarges the amplitude of cyclin/CDK oscillations. The corresponding limit cycle oscillations of the cell cycle in the presence or in the absence of PF loops are illustrated in the cyclin B/CDK1 vs. cyclin A/CDK2 plot (Figures 2F,H, where red curves correspond to deterministic limit cycle oscillations while black curves correspond to stochastic oscillations).

The results of stochastic simulations indicate that the robustness of the cell cycle dynamics toward random fluctuations in gene expression critically depends on the presence of multiple, redundant, PF loops at the different cell cycle phase transitions.

## Calcium dynamics at the edge between stochastic and deterministic behaviors

Depending on the conditions, intracellular Ca^2+^ dynamics can appear as very noisy or display rather regular oscillations (Dupont et al., 2016). Increase in cytosolic Ca^2+^ is initiated by a rise in the concentration of inositol 1,4,5-trisphosphate (IP_3_), a messenger that is synthesized in response to an external hormonal stimulation. When IP_3_ binds to specific receptors (IP_3_R) located in the endoplasmic reticulum (ER) membrane, these receptors release Ca^2+^ from the ER into the cytosol. Cytosolic Ca^2+^ itself regulates the activity of IP_3_R: low concentrations of Ca^2+^ stimulate the opening of the channels, while higher concentrations tend to close them. The positive feedback of cytosolic Ca^2+^ on its own release through IP_3_R, known as “Ca^2+^-induced Ca^2+^ release,” plays a crucial role in Ca^2+^ dynamics. It allows the occurrence of oscillations and permits the spatial coupling between IP_3_R, as the Ca^2+^ released by one IP_3_R stimulates the release of Ca^2+^ from a neighboring IP_3_-bound IP_3_R (Dupont et al., 2016).

**Figure 3:**
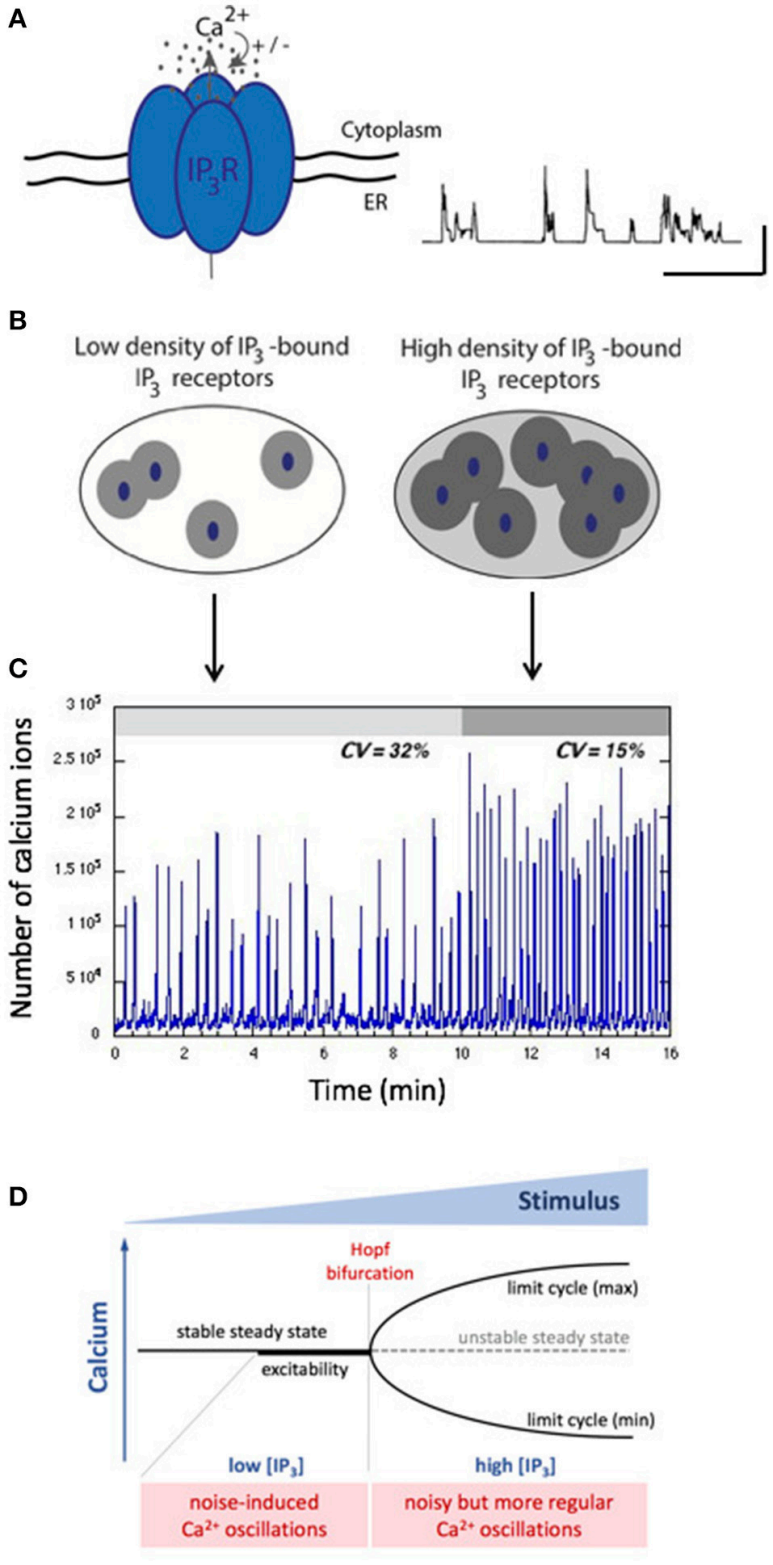
Stochastic vs. deterministic character of intracellular Ca^2+^ dynamics. **(A)** The IP_3_R that opens upon IP_3_ binding and is biphasically regulated by Ca^2+^ is the building block of intracellular Ca^2+^ dynamics. When isolated or clustered in small numbers, IP_3_R open randomly in a repetitive manner leading to localized, highly variables Ca^2+^ signals called “puffs.” The trace shows stochastic simulations of such Ca^2+^ puffs obtained as in Swillens et al. (1999). The horizontal and vertical bars represent 1 s and 100 nM, respectively. **(B)** Depending on the level of IP_3_, different numbers of IP_3_R participate to the cellular Ca^2+^ dynamics. When IP_3_ is low, few IP_3_R participate (left) while at high concentration of IP_3_, many channels participate, which increases the spatial coupling through cytosolic Ca^2+^ (right). **(C)** Gillespie's simulations indicate that two situations generate qualitatively different Ca^2+^ dynamics. For sub-threshold IP_3_ concentrations, noisy spiking (with a high coefficient of variation) arises. A small increase in IP_3_ concentration allows the passage of a threshold, in which case spiking corresponds to limit-cycle oscillations perturbed by noise. See Dupont et al. (2008) for details. **(D)** Schematic representation of the stochastic vs. deterministic character of Ca^2+^ dynamics in terms of a bifurcation diagram. Below the Hopf bifurcation point, when the system is excitable, stochastic resonance enhances the effect of random fluctuations. After the Hopf bifurcation point, the system is oscillatory, and noise perturbs the regular character of the oscillations.

When IP_3_ concentration is low, only a few IP_3_R have IP_3_ bound and can be active. As Ca^2+^ does not diffuse well in the cytoplasm because of heavy Ca^2+^ buffering, Ca^2+^ increases remain localized around clusters of IP_3_R. In consequence, cells display random repetitive localized Ca^2+^ increases called “Ca^2+^ puffs” (Keebler and Taylor, 2017). These puffs can be reproduced by stochastic simulations of closely packed IP_3_R, as illustrated in Figure 3A (Swillens et al., 1999; Thul et al., 2009). Following an increase in IP_3_ concentration, Ca^2+^ increases invade the whole cytosol and cells thus display repetitive sequences of cellular Ca^2+^ spikes, which appear more or less regular depending on the cell type and on the level of stimulation by the external hormone or by IP_3_ directly.

While some analyses conclude that the cellular Ca^2+^ dynamics is intrinsically stochastic at all stimulation levels (Skupin and Falcke, 2009; Thurley et al., 2014), it is also highly plausible that at the global level, Ca^2+^ oscillations correspond to a deterministic limit cycle perturbed by noise (Kummer et al., 2005; Dupont et al., 2008; Cao et al., 2014; Li et al., 2018). As schematized in Figure 3B, at low concentrations of IP_3_, only a few clusters of IP_3_R are active. Hence, little Ca^2+^ is released and spatial coupling is limited. In contrast, at high concentrations of IP_3_, many channels are active, allowing an efficient coupling between clusters of IP_3_R through Ca^2+^-induced Ca^2+^ release. The passage from a stochastic to a noisy deterministic regime upon increasing the level of stimulation was tested experimentally in noradrenaline-stimulated hepatocytes (Dupont et al., 2008). While sub-threshold stimulation levels induce either no response or highly irregular Ca^2+^ spikes, a tiny increase in stimulation allows oscillations to become much more regular (Figure 3C). The coefficient of variation estimated on ~30 time series decreases from 31 to 12%. Simulations suggest that this behavior can be explained by the passage from an excitable regime displaying stochastic resonance to an oscillatory regime following the crossing of the Hopf bifurcation point because of a slight increase in the concentration of IP_3_ (Dupont et al., 2008). Stochastic resonance is a well-known mechanism by which a system amplifies weak signals emitted by a noisy environment (Benzi et al., 1981). Fluctuations allowing stochastic resonance are due to the small number of IP_3_R clusters. A recent study (Keebler and Taylor, 2017) estimates that a HeLa cell, which has roughly the same size as a hepatocyte, has ~100 puff sites, in agreement with the low number of clusters used to simulate stochastic resonance-based Ca^2+^ spiking in hepatocytes.

## Increase of cellular noise due to calcium compartmentalization in cellular organelles

IP_3_-induced Ca^2+^ release from the ER is accompanied by Ca^2+^ fluxes in and out mitochondria. By entering in these organelles, Ca^2+^ activates the Krebs cycle and allows the coupling of ATP supply with energy demand (Duchen, 1999). Ca^2+^ enters into mitochondria via the mitochondrial Ca^2+^ uniporter (MCU), whose activity depends on Ca^2+^ and on the mitochondrial membrane potential. Ca^2+^ efflux occurs through Na^+^/Ca^2+^ and H^+^/Ca^2+^ exchangers. At each cytosolic Ca^2+^ spike, mitochondria sequester some of the Ca^2+^ released from the ER into the cytosol through the IP_3_ receptors (Ishii et al., 2007; Wacquier et al., 2016). Most of these Ca^2+^ ions then bind to buffers that are present in large concentrations inside mitochondria. After the return of the cytosolic Ca^2+^ concentration to its basal value, mitochondria start releasing their Ca^2+^, which progressively dissociates from buffers. By releasing Ca^2+^ in the cytoplasm during the interspike interval, mitochondria play an active role in cellular Ca^2+^ dynamics: they control the frequency of the Ca^2+^ spikes by regulating the rate of IP_3_ receptor reactivation.

**Figure 4:**
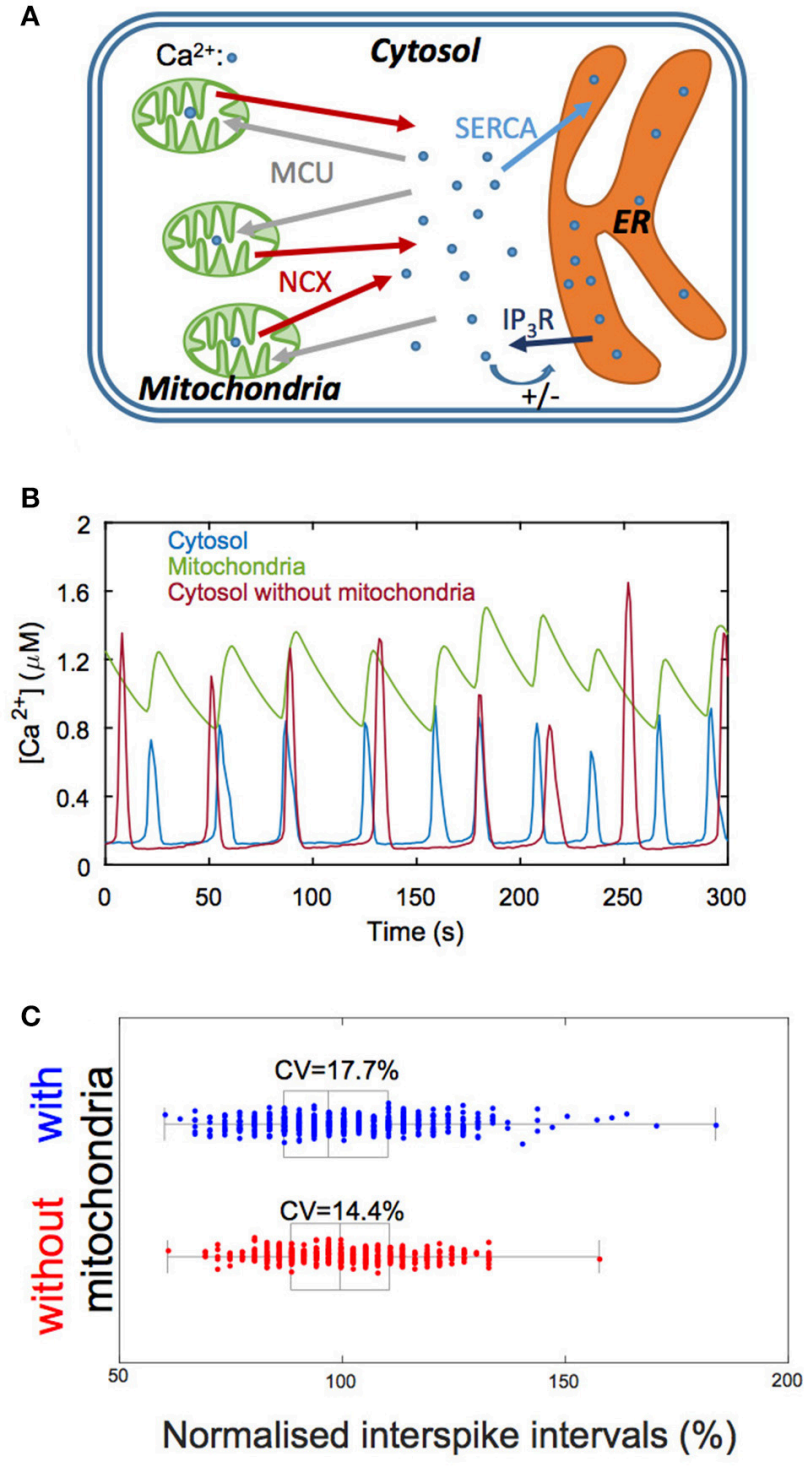
Involvement of mitochondrial Ca^2+^ fluxes in simulations of Ca^2+^ oscillations. **(A)** Schematic representation of the Ca^2+^ exchanges processes between the three main cellular Ca^2+^ compartments: the cytosol, the mitochondria and the endoplasmic reticulum (ER). IP_3_R: IP_3_ receptor; MCU: mitochondrial Ca^2+^ uniporter; NCX: Na^+^/Ca^2+^ exchanger; SERCA: sarco- or endoplasmic reticulum Ca^2+^ ATPase. **(B)** Numerical simulations of Ca^2+^ oscillations in a model including mitochondrial Ca^2+^ fluxes (blue and green traces) and in a model that does not consider this organelle (red trace). Given the small volume of mitochondria, mitochondrial Ca^2+^ fluxes are quite irregular, which impacts on the variability of the interspike interval. Note, however, that the amplitude of the oscillations is stabilized in the presence of mitochondria. **(C)** Statistical analysis of the interspike interval when considering (blue) or neglecting (red) mitochondrial Ca^2+^ fluxes. Boxes indicate the first quartiles of the distributions and bars inside the box, the median values. Interspike intervals are normalized with respect to the corresponding deterministic value. See Wacquier et al. (2016, 2017) for details.

Mitochondria are small organelles containing a limited number of Ca^2+^ ions and can thus be seen as potential sources of randomness in Ca^2+^ dynamics. We investigated this question by performing Gillespie's simulations of a model of Ca^2+^ dynamics taking mitochondria into account (Wacquier et al., 2016, 2017). The model (Figure 4A) describes both Ca^2+^ dynamics and mitochondrial metabolism as these processes closely interact. It is visible (Figure 4B) that mitochondrial Ca^2+^ dynamics is more affected by noise than cytoplasmic Ca^2+^ dynamics: the baseline and shape of the oscillations in mitochondria (green curve) are more variable than in the cytosol (blue curve). Moreover, this noise strongly impacts on the regularity of cytosolic Ca^2+^ oscillations. Simulations indicate that eliminating the mitochondrial fluxes always decreases the coefficient of variation (CV) of the interspike interval (Figure 4C). This decrease in CV can be as large as 20% for realistic values of cell and mitochondrial volume (100 and 7.3 μm^3^ for the total and mitochondrial volumes, respectively).

This computational prediction thus suggests that the coupling between Ca^2+^ signaling and mitochondrial metabolism occurs at the expense of the regularity of the stimulation-induced cytosolic Ca^2+^ spikes.

## Noise as a trigger to initiate embryonic cell fate determination

**Figure 5:**
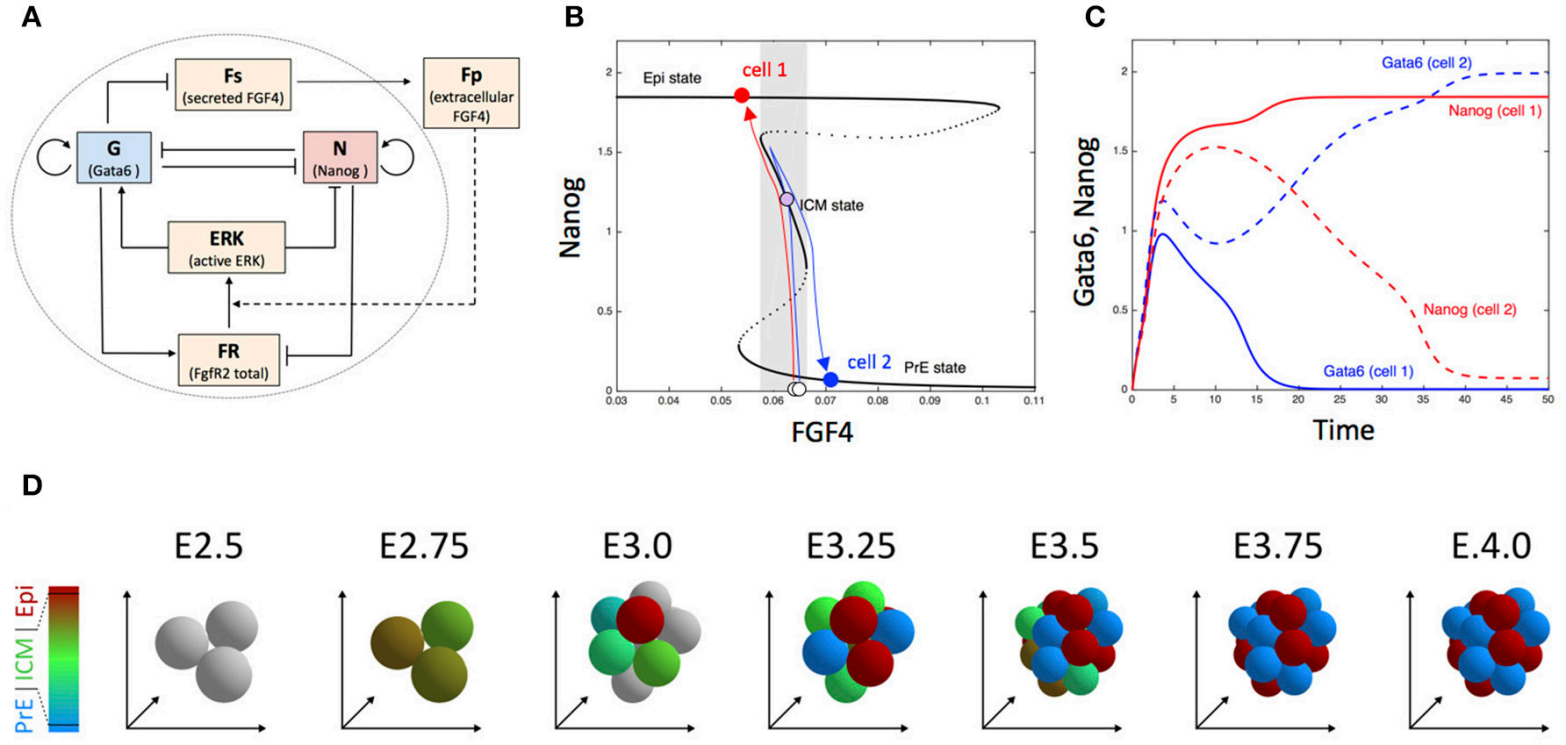
Dynamics of embryonic cell fate determination. **(A)** Scheme of the gene regulatory network. **(B)** Bifurcation diagram showing the steady state of Nanog as a function of FGF4. The blue and red arrows depict the trajectory of two cells (for more details, see De Mot et al., 2016). **(C)** Time evolution of Nanog and Gata6 in the two-cell model. **(D)** Snapshots showing the simulated the evolution of embryonic cells from the 3 inner cells stage in the 3D model including cell division. Outer cells (trophectoderm) are not considered. Cells are colored according to the proportion of Nanog (red) and Gata6 (blue), with gray indicating blastomers with very low Nanog and Gata6 concentrations. See Tosenberger et al. (2017) for details.

Cell specification in early mammalian blastocysts is governed by interactions between transcription factors, modulated by extracellular signaling. Although cell differentiation is highly organized, both spatially and temporally, noise is involved in this process and extensive expression heterogeneities among cells precede the emergence of lineage (Chazaud and Yamanaka, 2016). In mice, the second differentiation process, which corresponds to the specification of the Inner Cell Mass (ICM) cells into Primitive Endoderm (PrE) and Epiblast (Epi) cells, can be described as the evolution toward one of the multiple steady states of the gene regulatory network schematized in Figure 5A (Bessonnard et al., 2014). Nanog and Gata6 are the transcription factors necessary to produce Epi and PrE cells, respectively. Their mutual inhibition is coupled to auto-activation. Moreover, Gata6 activates the ERK pathway while Nanog inhibits it. Besides, cells communicate through the secretion of Fgf4, which is inhibited by Gata6. Thus, Nanog-expressing cells (Epi) stimulate the Erk pathway in Gata6-expressing cells (PrE). Modeling suggests that this complex network of interactions allows for the existence of three stable steady states in the levels of expression of transcription factors, as shown in Figure 5B (De Mot et al., 2016; Tosenberger et al., 2017). Each steady state corresponds to a given cell fate: PrE (high Gata6, high Erk, low Nanog), Epi (high Nanog, low Erk, low Gata6) and undifferentiated ICM (intermediate Nanog, Gata6, and Erk). However, observations in developing embryos show that the evolution toward one or the other cell fate is not fully deterministic, as at the 64 cell stage, Epi, and PrE cells are arranged in a mixed and seemingly random fashion, referred to as salt-and-pepper pattern (Kang et al., 2013).

Dynamical simulations of a system of four ODE describing the cellular regulatory network shown in Figure 5A suggests that noise on extracellular Fgf4 plays a key role in the establishment of the salt-and-pepper pattern, but also in the specification process itself (Bessonnard et al., 2014; De Mot et al., 2016). This dynamical process is represented in Figures 5B,**C** for the simplified case of two differentiating cells. When the specification process begins, Gata6, and Nanog are weakly expressed. As the level of extracellular Fgf4 is intermediate, cells evolve toward the middle branch of the bifurcation diagram, i.e., the ICM state where both transcription factors are co-expressed. In the extracellular medium, Fgf4 decreases due to its degradation. The two cells then move toward the left part of the bifurcation diagram. If, because of external noise, one of the cells feels a slightly lower concentration of Fgf4 than the other cell (cell 1 in the schematic representation shown in Figure 5B), this cell will reach the end of the intermediate branch before the other one and will “jump” to the Epi branch. In consequence, the level of Gata6 decreases in cell 1 and its secretion of Fgf4 increases. The resulting increase in extracellular Fgf4 induces the transition of cell 2 toward the PrE state, while cell 1 remains on the Epi state because the branch is stable over a wide range of Fgf4 concentrations.

When simulated for a realistic number of cells that can also divide (Tosenberger et al., 2017), the same computational model can account for the specification of the inner cells of the embryo into the different cell types, which are moreover arranged in a salt-and-pepper pattern as observed *in vivo* (Figure 5D). Indeed a given cell tends to be surrounded by cells of the other type (as quantified in Figure 3D in Tosenberger et al., 2017). Thus, noise on external Fgf4—that likely originates from restricted diffusion in the compact embryo—can initiate cell fate specification, which then occurs in a self-organized process thanks to the interplay between the gene regulatory network and extracellular signaling through Fgf4. Other computational approaches emphasize the importance of the interplay between multistability and noise in early mammalian development (Krupinski et al., 2011; Ohnishi et al., 2014; Nissen et al., 2017).

Additional sources of heterogeneity include the variability in the duration of cell division, unequal partition of the molecules in the daughter cells after cell division, and noise on the initial concentration of the key regulatory factors (namely Nanog and Gata6). Sensitivity analyses confirmed that the proposed specification mechanism is robust with respect to these factors. Indeed, the proportions of the differentiated cell types and the salt-and-pepper pattern remain unchanged in presence of a moderate amount of these extrinsic sources of noise (Tosenberger et al., 2017).

## Concluding remarks

Biological systems are inevitably subject to noise. Both intrinsic and extrinsic sources of noise affect cellular dynamics, so that genetically identical cells in the same environment can behave differently from one to another. These differences are manifested by the heterogeneity in the level of gene expression (Elowitz et al., 2002) or the progressive desynchronization of oscillations (Elowitz and Leibler, 2000). Noise is thus often seen as a nuisance, but it can also have constructive roles. We have illustrated here how modeling-based approaches provide insights on the effect of noise in a variety of selected cellular systems including oscillatory networks, signaling pathways, and developmental processes.

### Noise in oscillatory processes

Circadian rhythms must ensure a proper adaptation of the organisms to cyclical environmental conditions. The cell division cycle must be tightly regulated to guarantee an adequate density- and environment-dependent growth. We may thus expect that noise reduction mechanisms are implemented in the molecular circuitry of these complex cellular systems. Mathematical modeling allow to explore regulatory network features that may contribute to the robustness of oscillations. Thus stochastic simulations of models for circadian clocks and for the cell cycle highlight how the robustness of oscillations is affected by various factors such as positive feedback loops, transcriptional mechanisms, nonlinear kinetics, or external periodic forcing. Coupling transcription-translation cycle to protein phosphorylation cycle may also contribute to the robustness of circadian oscillations (Zwicker et al., 2010). Besides the design of individual cellular oscillators, the coupling between cells constitutes an additional strategy to cope with noise. The idea that robust circadian oscillations may emerge from the coupling between sloppy individual clocks, predicted by theoretical modeling, appears to be supported by single cell recording in both dispersed and coupled cells. However, further investigations are needed to fully clarify the relative contributions of the molecular network architecture, the kinetic nonlinearities, the intercellular coupling and the different sources of noise in enhancing the robustness of biological oscillators.

During the last years, microRNAs (miRNAs) were identified as a potential additional source of robustness against molecular fluctuations. MicroRNAs are noncoding RNA molecules of 20–30 nucleotides. They bind to the 3′ UTR of messenger RNA which represses protein synthesis by targeting the corresponding messenger RNA for degradation and/or by inhibiting its translation (Bartel, 2009). Besides the key role of miRNAs for the down-regulation of protein expression (Guo et al., 2010), it was shown that miRNAs can induce thresholds in protein synthesis (Mukherji et al., 2011). Moreover, they are often involved in feed-forward regulations with their target genes, allowing an increase in the robustness of protein expression toward molecular noise (Osella et al., 2011). MicroRNAs provide a new layer in the regulation of protein expression that can confer robustness of gene expression (Ebert and Sharp, 2012). In addition, numerous miRNAs have been identified to play key roles in the dynamics of regulatory networks driving the cell cycle (Bueno and Malumbres, 2011) or the circadian clock (Cheng et al., 2007). In that framework, a theoretical model indicates that the presence of miRNA embedded in a negative feedback loop, which can characterize the regulatory structure of the circadian rhythms, could enhance the time delay of the oscillations and favor the oscillations of large amplitude (Gérard and Novak, 2013).

### Noise in signaling

One of the key characteristics of Ca^2+^ oscillations is that their frequency increases with the level of external stimulation, thus leading to the transformation of an analog signal (the agonist concentration) into a digital one (the frequency of the repetitive Ca^2+^ spikes). It is well known that frequency-encoded signals are more resistant to noise than amplitude-coded ones (Rapp et al., 1981). The question however arises as how frequency-encoded signals might be affected by significant stochastic variations in the average interspike interval, observed in many cell types. A detailed analysis of the stimulus-frequency relationship in individual cells has shown that changes in the extracellular stimulus intensity are encoded by fold changes in the average interspike interval, this fold change being similar for each cell despite the large CV on intervals within each cell and despite the large variability in average interspike interval in individual cells (Thurley et al., 2014). Interestingly, such fold change in response after simulation increase has been reported for other signaling pathways, as for example the nuclear concentration of the NF-κB transcription factor (Lee et al., 2014).

### Noise in development

Many developmental processes are based on spatial information (such as gradients of morphogens) but in some cases stochasticity holds a key role in cell fate determination. Stochasticity does not mean that everything is random. A stochastic component serves as a source of heterogeneity, often required to lead cells in one or another developmental pathway. These pathways are typically associated with alternative steady states. If two or more stable steady states coexist in the same environmental conditions (bi/multi-stability), a small difference in the initial conditions of different cells can be sufficient to induce their evolution toward different steady states (fates), as exemplified in the embryonic differentiation system described above. To be reliable, the differentiation mechanism should also prevent spontaneous, noise-induced switches between cell fates and should lead to well-proportioned cells in each fate. This can be achieved by cooperative roles of multiple regulatory feedbacks (Pfeuty and Kaneko, 2016).

Differentiation based on bi/multi-stable systems generates permanent, irreversible cell fates. In other cases, differentiated states are only transient. This is the case for example of the “competent” state in *Bacillus subtilis*, a state characterized by the capability for the bacteria to uptake DNA from the environment. A model based on the gene regulatory network explains the spontaneous and transient entry into competence in term of excitability and noise-driven excursion in the phase space (Süel et al., 2006). The model highlights the importance of both positive and negative feedback loops. Whereas the positive feedback controls the frequency at which cells become competent, the negative circuit is crucial for the exit from the competent state. Bypassing the negative circuit is thus predicted to “stabilize” the cells in the competent state. This prediction was verified experimentally (Süel et al., 2006). Such mechanism may not be limited to bacteria. A similar mechanism was postulated to occur in mammalian embryonic stem cell differentiation (Kalmar et al., 2009).

Finally, it should be noted that, although treated separately, the different processes discussed here are, in reality, not independent of each other. Mutual coupling between the circadian clock and the cell cycle (Feillet et al., 2015), circadian regulation of cell differentiation (Brown, 2014), or signaling-dependent cell fate decision (Sonnen and Aulehla, 2014) are now well established. Fluctuations in the environment or generated at the level of a given process are then expected to be transmitted to the coupled processes. Regulatory mechanisms may thus have evolved to counteract the undesired propagation of noise, for example from the cell cycle to the circadian clock, as suggested by the theoretical study of Paijmans et al. (2016). Similarly, as cell fate decision is critical for the development and survival of the organism, signaling must be able to reliably filter noise from actual environmental changes. For some specification steps, however, the transmission of stochasticity may be advantageous. It was also speculated that (dephased) circadian oscillators could constitute a source of heterogeneity for stem cell, allowing them to optimally respond to various signals (Brown, 2014).

### Conclusion

The dynamics of biological processes results from the interplay between the hard-wired architecture of regulatory circuits and the multiple sources of noise. Deterministic and stochastic modeling approaches constitute valuable tools to interpret experimental observations and to make sense of the biological complexity. The underlying bistability, dependent on positive feedback loops, and its contribution to the robustness of the cell cycle or the phenomenon of stochastic resonance revealed by models of Ca^2+^ signaling are features that would not be predicted by sheer intuition. Even less predictable is the behavior of interacting cell populations in the presence of noise. Noise-induced synchronization of circadian clocks and noise-induced cell differentiation are properties that were revealed by modeling. However many questions remain open. Various aspects such as for example, the contribution of miRNA in the robustness of oscillatory processes or the relative importance of the different sources of noise involved in cell fate determination still need to be investigated. The path, initiated by Delbrück in 1940, has beautiful days ahead.

## Author contributions

DG, CG, and GD wrote the core of the paper. DG, CG, BW, AW, AT, AG, and GD contributed to ideas and revised the paper.

### Conflict of interest statement

The authors declare that the research was conducted in the absence of any commercial or financial relationships that could be construed as a potential conflict of interest.
