# Supplementary material for: Modeling-Based Investigation of the Effect of Noise in Cellular Systems
Source: Front Mol Biosci. 2018 Apr 12;5:34. doi: 10.3389/fmolb.2018.00034 (PMC5907451; doi:10.3389/fmolb.2018.00034)
Supplement: Supplementary file 1 [file Presentation1.PDF]

SUPPLEMENTARY INFORMATION

Modeling-based investigation of the effect of noise in cellular systems

Didier Gonze[1], Claude Gérard[2], Benjamin Wacquier[1], Aurore Woller[1], Alen Tosenberger[1], Albert Goldbeter[1], Geneviève Dupont[1]

(1) Unité de Chronobiologie Théorique, Faculté des Sciences, Université Libre de Bruxelles (ULB), Brussels, Belgium.
(2) de Duve Institute, Université Catholique de Louvain (UCL), Brussels, Belgium

Stochastic simulations

Molecular noise results from the low number of molecules and the probabilistic character of the (bio)chemical reactions. The Gillespie algorithm is a standard and rigorous way to simulate stochastic (bio)chemical systems [3]. In Figs. 1-4, molecular noise was simulated with this algorithm. A certain propensity is associated to each reaction. These propensities can be derived from the macroscopic kinetic rates and can be converted into probabilities. At each time step, the algorithm determines (1) the reaction that takes place according to their probabilities, and (2) the time interval to the next reaction (which also depends on the propensities of the reactions). At each time step, the numbers of molecules of the different reacting species as well as the reaction propensities are updated. In this approach, a parameter, referred to as the system size and denoted Ω, is used to convert the concentrations into the numbers of molecules. This parameter has the unit of a volume and allows to control the number of molecules in the system, and thereby the level of molecular noise. The original Gillespie algorithm is rigorous, but running such stochastic simulations might be CPU-consuming (even for relatively small systems, millions of reactions may occur in a limited time window). This is why alternative, approximate, or hybrid approaches have been proposed (for a review of these methods, see [10]). Another way to limit memory and CPU requirements is to use compact, nonlinear equations (i.e., Michaelis-Menten or Hill functions) to compute the reaction propensities [11, 9].

Example: Stochastic model for the circadian clock

As an example of the application of the Gillespie algorithm, we show here how the Goodwin-like model for the circadian clock was converted into a stochastic version and simulated with the Gillespie algorithm. The reaction scheme involves three "clock" variables, namely X, Y, and Z, which form the core negative feedback loop responsible for the circadian oscillations, and an output component, V (which can be regarded as a circadian-controlled neurotransmitter).

$$\begin{aligned} &\xrightarrow{k_1 f(Z)} \mathrm{X} \xrightarrow{k_2} \\ &\xrightarrow{k_3} \mathrm{Y} \xrightarrow{k_4} \\ &\xrightarrow{k_5} \mathrm{Z} \xrightarrow{k_6} \\ &\xrightarrow{k_7} \mathrm{V} \xrightarrow{k_8} \end{aligned} \tag{1}$$

The corresponding deterministic equations write [6]:

$$\begin{aligned} \frac{dX}{dt} &= k_1 \frac{K_i^n}{K_i^n + Z^n} - k_2 \frac{X}{K_x + X} \\ \frac{dY}{dt} &= k_3 X - k_4 \frac{Y}{K_y + Y} \\ \frac{dZ}{dt} &= k_5 Y - k_6 \frac{Z}{K_z + Z} \\ \frac{dV}{dt} &= k_7 X - k_8 \frac{V}{K_v + V} \end{aligned} \tag{2}$$

Parameter values used in the simulations shown in Fig. 1 are: $k_1 = 0.7$ nM/h, $K_i = 1$ nM, $n = 4$, $k_2 = 0.35$ nM/h, $K_x = 1$ nM, $k_3 = 0.7$ /h, $k_4 = 0.35$ nM/h, $K_y = 1$ nM, $k_5 = 0.7$/h, $k_6 = 0.35$ nM/h, $K_z = 1$ nM, $k_7 = 0.35$ /h, $k_8 = 1$ nM/h, $K_v = 1$ nM [6]. To account for the cell-cell variability of the period, parameters k_i are multiplied by a scaling factor σ (identical for all k_i of a given cell), so that only the period is affected [6]. Moreover, since the period of the coupled oscillators system slightly shifts towards values larger than the average period of the individual oscillators [6], the rescaling factors are chosen so that the synchronized oscillators display a period of 24h. This allows the comparison of the robustness (i.e. the standard deviation of the periods) between isolated and coupled oscillators.

This deterministic model is then converted into a set of reactions, for which the propensities can be derived from the deterministic kinetic rates (Table S1). Since the stochastic version requires a description in term of numbers of molecules (rather than concentrations), the variables and kinetic parameters need to be converted accordingly.

No	Reaction	Propensity	No	Reaction	Propensity
1	$\rightarrow X$	$w_1 = k'_1 \Omega \dfrac{K'^n_i}{K'^n_i + Z^n}$	5	$\rightarrow Z$	$w_5 = k_5 Y$
2	$X \rightarrow$	$w_2 = k'_2 \dfrac{X}{K'_x + X}$	6	$Z \rightarrow$	$w_6 = k'_6 \dfrac{Z}{K'_z + Z}$
3	$\rightarrow Y$	$w_3 = k_3 X$	7	$\rightarrow V$	$w_7 = k_7 V$
4	$Y \rightarrow$	$w_4 = k'_4 \dfrac{Y}{K'_y + Y}$	8	$V \rightarrow$	$w_8 = k'_8 \dfrac{V}{K'_v + V}$

Table S1: Stochastic version of the circadian clock model. In this version, the variables are expressed in numbers of molecules. Parameter values have thus to be adapted accordingly: $k'_1 = k_1\Omega$, $K'_i = K_i\Omega$, $K'_x = K_x\Omega$, $k'_4 = k_4\Omega$, $K'_y = K_y\Omega$, $k'_6 = k_6\Omega$, $K'_z = K_z\Omega$, $k'_8 = k_8\Omega$, $K'_v = K_v\Omega$. See [5, 7] for details.

References for the figures presented in the paper

References to the original papers where model equations, simulation procedures and additional figures can be found are given in Table S2.

Figure	Description	Original references
1	Circadian clock	Gonze et al, 2002 [5]
		Gonze et al, 2006 [7]
2	Cell cycle (toy model)	Gonze and Hafner, 2010 [8]
	Cell cycle (mammalian model)	Gérard et al, 2012 [4]
3	Calcium	Dupont et al., 2008 [2]
4	Calcium	Wacquier et al. 2016 [13]
		Wacquier et al., 2017 [14]
5	Cell fate determination	De Mot et al, 2016 [1]
		Tosenberger et al, 2017 [12]

Table S2: References for the figures presented in the paper. In Figs. 1-4, molecular noise was simulated with the Gillespie algorithm as described above. In Fig. 5D, the gene regulatory dynamics is treated deterministically, but extrinsic sources of noise include variability in Fgf4 perception, asynchrony in cell division, random movement of the cells, and unequal partition of the molecules in the daughter cells.

References

[1] De Mot L, Gonze D, Bessonnard S, Chazaud C, Goldbeter A, Dupont G (2016) Cell Fate Specification Based on Tristability in the Inner Cell Mass of Mouse Blastocysts. *Biophys.* J. 110: 710-722.

[2] Dupont G, Abou-Lovergne A, Combettes L (2008) Stochastic aspects of oscillatory Ca^{2+} dynamics in hepatocytes. *Biophys. J.* 95: 2193-2202.

[3] Gillespie DT (1977). Exact Stochastic Simulation of Coupled Chemical Reactions. *J Phys Chem* 81: 2340-2361.

[4] Gérard C, Gonze D, Goldbeter A (2012) Effect of positive feedback loops on the robustness of oscillations in the network of cyclin-dependent kinases driving the mammalian cell cycle. *FEBS J* 279:3411-31.

[5] Gonze D, Halloy J, Goldbeter A (2002) Robustness of circadian rhythms with respect to molecular noise. *Proc Natl Acad Sci USA*. 99:673-8.

[6] Gonze D, Bernard S, Waltermann C, Kramer A, Herzel H (2005) Spontaneous synchronization of coupled circadian oscillators. *Biophys J.* 89:120-9.

[7] Gonze D, Goldbeter A (2006) Circadian rhythms and molecular noise. *Chaos* 16:026110.

[8] Gonze D, Hafner M (2010). Positive feedbacks contribute to the robustness of the cell cycle with respect to molecular noise. *Advances in the Theory of Control, Signals, and Systems, LNCIS*, pp. 283-295.

[9] Gonze D, Abou-Jaoudé W, Ouattara DA, Halloy J (2011) How molecular should your molecular model be? On the level of molecular detail required to simulate biological networks in systems and synthetic biology. *Methods Enzymol* 487:171-215.

[10] Pahle J (2009) Biochemical simulations: stochastic, approximate stochastic and hybrid approaches. *Brief Bioinform.* 10:53-64.

[11] Rao C, Arkin A (2003) Stochastic chemical kinetics and the quasi steady-state assumption: application to the Gillespie algorithm *J. Chem. Phys* 118:4999-5010.

[12] Tosenberger A, Gonze D, Bessonnard S, Cohen-Tannoudji M, Chazaud C, Dupont G (2017) A multiscale model of early cell lineage specification including cell division. *NPJ Systems Biology and Applications* 3:16.

[13] Wacquier B, Combettes L, Tran Van Nhieu G, Dupont G (2016) Interplay between intracellular Ca^{2+} oscillations and Ca^{2+}-stimulated mitochondrial metabolism. *Scient. Rep.* 6: 19316.

[14] Wacquier B, Romero Campos HE, Gonzalez-Velez V, Combettes L, Dupont G (2017) Mitochondrial Ca2+ dynamics in cells and suspensions. *FEBS J.* 284: 4128-4142.
